# A combined cuff electrode array for organ-specific selective stimulation of vagus nerve enabled by Electrical Impedance Tomography

**DOI:** 10.3389/fmedt.2023.1122016

**Published:** 2023-04-17

**Authors:** Enrico Ravagli, Jeffrey Ardell, David Holder, Kirill Aristovich

**Affiliations:** ^1^ Department of Medical Physics and Biomedical Engineering, University College London, London, United Kingdom; ^2^ Department of Medicine, Cardiac Arrhythmia Centre, University of California Los Angeles, Los Angeles, CA, United States

**Keywords:** electrical impedance tomography, EIT, electrode array, neuromodulation, selective stimulation, vagus nerve

## Abstract

Previously developed spatially-selective Vagus Nerve Stimulation (sVNS) allows the targeting of specific nerve fascicles through current steering in a multi-electrode nerve cuff but relies on a trial-and-error strategy to identify the relative orientation between electrodes and fascicles. Fast Neural Electrical Impedance Tomography (FN-EIT) has been recently used for imaging neural traffic in the vagus nerves of pigs in a cross-correlation study with sVNS and MicroCT fascicle tracking. FN-EIT has the potential for allowing targeted sVNS; however, up to now, stimulation and imaging have been performed with separate electrode arrays. In this study, different options were evaluated *in-silico* to integrate EIT and stimulation into a single electrode array without affecting spatial selectivity. The original pig vagus EIT electrode array geometry was compared with a geometry integrating sVNS and EIT electrodes, and with direct use of sVNS electrodes for EIT imaging. Modelling results indicated that both new designs could achieve image quality similar to the original electrode geometry in all tested markers (e.g., co-localisation error <100 µm). The sVNS array was considered to be the simplest due to the lower number of electrodes. Experimental results from testing evoked EIT imaging of recurrent laryngeal activity using electrodes from the sVNS cuff returned a signal-to-noise ratio similar to our previous study (3.9 ± 2.4 vs. 4.1 ± 1.5, *N* = 4 nerves from 3 pigs) and a lower co-localisation error (≈14% nerve diameter vs. ≈25%, *N* = 2 nerves from 2 pigs). Performing FN-EIT and sVNS on the same nerve cuff will facilitate translation to humans, simplify surgery and enable targeted neuromodulation strategies.

## 1. Introduction

The new field of neuromodulation aims to treat diseases and alleviate conditions through the use of electrical stimulation of neural tissue rather than drugs or radiation (1). One of the main targets of bioelectronic medicine is the vagus nerve, a cranial nerve and part of the autonomic nervous system; since it provides innervation to several visceral organs in the thorax and abdomen, as well as the larynx, it is a prime candidate to modulate the activity of multiple systems from a single point of action (2).

Traditionally, vagus nerve stimulation (VNS) is performed by implantation at the cervical level of a simple nerve cuff embedding two electrodes, which stimulate the entire nerve (2). While some selectivity can be achieved with respect to the type of nerve fibres, for example adjusting stimulation parameters (3), full VNS still usually leads to the presence of off-target effects which affect organs other than the desired target. The recent development of spatially-selective VNS (sVNS) moves toward avoidance of off-target effects by employing a nerve cuff embedding two circumferential electrode arrays (4); current is delivered between pairs of electrodes occupying the same “o’clock” position in the two rings, thus steering the flow of charge toward a specific portion of the cross-sectional area of the nerve and toward specific nerve fibre bundles, i.e., fascicles. sVNS has been optimised in simulation and tested experimentally in sheep (4) and pigs (5), showing positive results in selective modulation of cardiac function, pulmonary function, and larynx activation.

One of the current drawbacks of sVNS is that it still relies on trial-and-error identification of the orientation of organ-specific sites in relation to the electrode pairs on the nerve cuff. To overcome this issue, Fast Neural Electrical Impedance Tomography (FN-EIT) (6) offers a possible tool to localise organ-related neural traffic over the cross-sectional area of the nerve and drive targeted sVNS. This technique, which works by imaging the bulk impedance changes generated by travelling action potentials, has been previously developed and optimised for evoked activity in the rat sciatic nerve (7–10) and was recently translated by our group to imaging spontaneous neural traffic in the vagus nerve of pigs (5). The majority of FN-EIT research has been performed with the ScouseTom EIT system (11) which was designed specifically for neural applications of EIT and features true parallel sampling on all channels, 24 bit vertical resolution and 100 kHz sampling rate. In the previous work in pigs (5), we employed two separate neural cuffs. FN-EIT was undertaken at the frequency of 6 kHz, with a single ring of 14 electrodes, each 0.35 × 1.5 mm. sVNS was undertaken with a second cuff containing two similar rings, with 14 electrodes 0.35 × 3 mm spaced 3.1 mm apart, with successive trial and error stimulation of a pair of electrodes in the same radial position on the two cuffs. This necessitated co-localisation of imaging and stimulation at different locations. All electrode arrays in our previous sVNS and EIT research were manufactured using the same process and PEDOT-coated to lower electrode-tissue contact impedance(12); typical impedance values ranged between 300–600 Ω, compared with transfer impedance values across the tissue of ≈500–2000 Ω.

The aim of the present work was to develop a single nerve cuff with an electrode array capable of both stimulation and imaging. This would be less invasive and offer a fixed known relation between stimulating and FN-EIT imaging electrodes. This could allow to first perform FN-EIT, and then perform targeted sVNS based on EIT information, thus enabling more accurate neuromodulation of the vagus nerve. In addition, further developments are possible that would allow sensing with FN-EIT to be performed with improved speed (13, 14). As such, the specific purpose of this work was to assess the feasibility and ideal geometry of a combined nerve electrode array cuff for performing both sVNS and EIT of the cervical vagus nerve, while organ-specific selective VNS was demonstrated in previous works (4, 5). Specific questions we aimed to answer by the end of this project were:

• Is it possible to develop a combined sVNS/EIT nerve cuff without compromising on stimulation or imaging features?
• What is the simplest geometry for a combined sVNS/EIT cuff?

## 2. Methods

### 2.1. Experimental design

We started this study by comparing *in-silico* the imaging quality of the existing nerve EIT cuff for pig cervical vagus (5) with candidate designs which integrated sVNS electrodes.

Optimal geometry for sVNS of the vagus nerve was previously identified by our group (4) and thus we aimed to leave it unchanged. This first design choice led to the following options for adding EIT imaging capabilities:

1. Use one ring of electrodes from the sVNS cuff directly for EIT imaging. In principle, this could yield good FN-EIT images. The difference would be use of electrodes with an increased length of 3.0 mm compared to that previously length of 1.5 mm. FN-EIT and sVNS are not intended to be performed concurrently and so there is no downside to this option.
2. Embed an additional ring of electrodes to be used for EIT in between the sVNS electrode rings, compatible with the fabrication capabilities of our nerve cuff manufacturing process (12).

We tested both options, leading to a comparison between:

• FN-EIT with the original EIT cuff from our prior study
• FN-EIT with the longer sVNS electrodes
• FN-EIT with the new “Intercalated” geometry

Simulations were performed with all candidate designs to assess image quality comparatively. Perturbations to background conductivity were implemented to generate realistic voltage data. Typical non-ideal conditions of EIT measurements were then applied to make the simulated data even more realistic, such as adding Gaussian noise with amplitude typical of experimental recordings, and removing a percentage of traces to simulate missing electrode data. Objective metrics of magnitude, spatial accuracy and shape conformity were used to evaluate the quality of images reconstructed from simulated data across designs.

The simplest geometry emerging from our *in-silico* assessment was tested experimentally in the course of an ongoing set of neuromodulation experiments on a pig model, recording impedance changes and performing EIT imaging at the level of the cervical vagus of neural traffic evoked from the recurrent laryngeal branch.

### 2.2. Modelling and simulation

**Figure 1:**
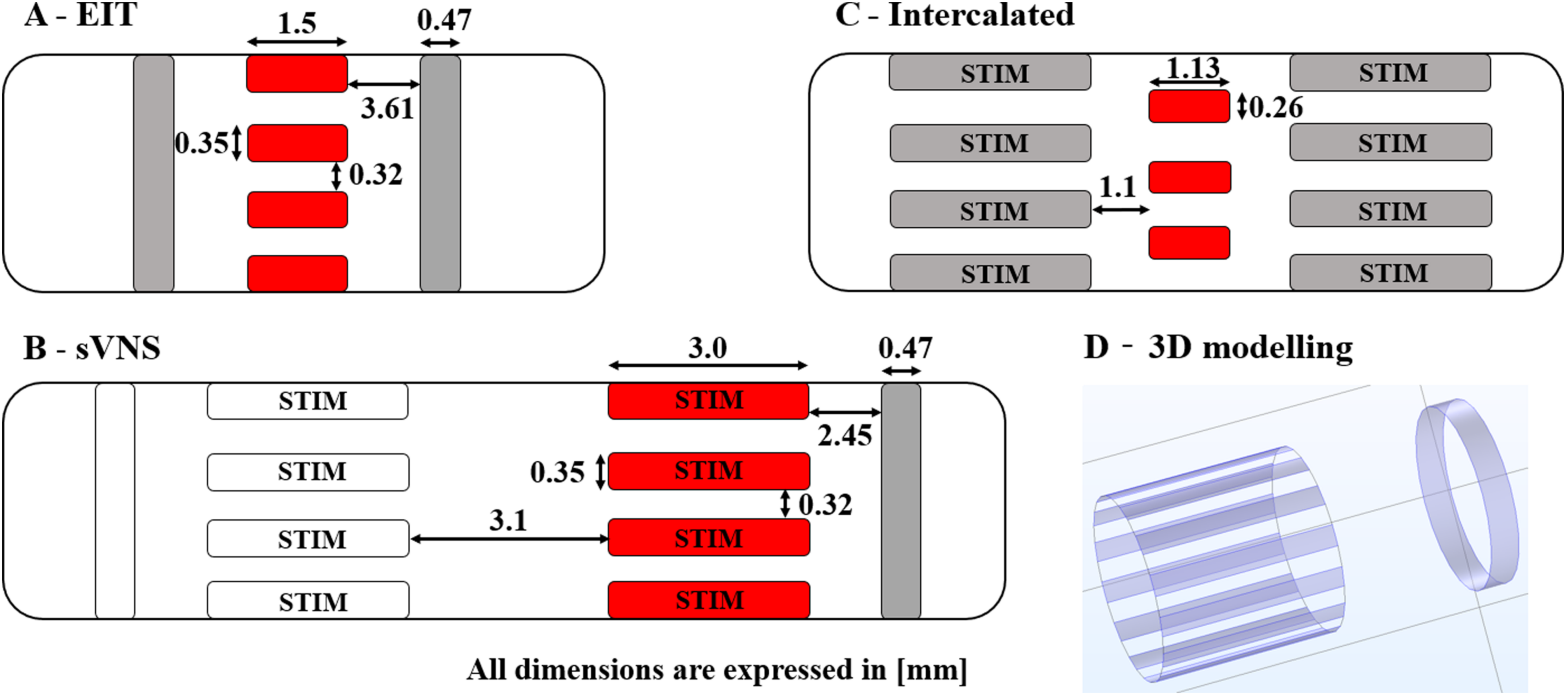
Diagrams of 3D electrode geometries tested in simulation with dimensions. (**A**) Original EIT array. (**B**) Original sVNS array. (**C**) Intercalated array. (**D**) Example of 3D electrode positions for the the sVNS design. Red pads indicate electrodes used for EIT current injection; grey pads electrodes are used as EIT voltage measurement reference. Electrode which are also used for selective stimulation are marked as “STIM”.

The original pig EIT nerve cuff design (Figure 1A) was compared with the sVNS design (Figure 1B), used for EIT imaging in single-ring configuration, and with the Intercalated design (Figure 1C), consisting of EIT electrodes placed in a ring configuration at equal distance from the sVNS electrodes.

**Figure 2:**
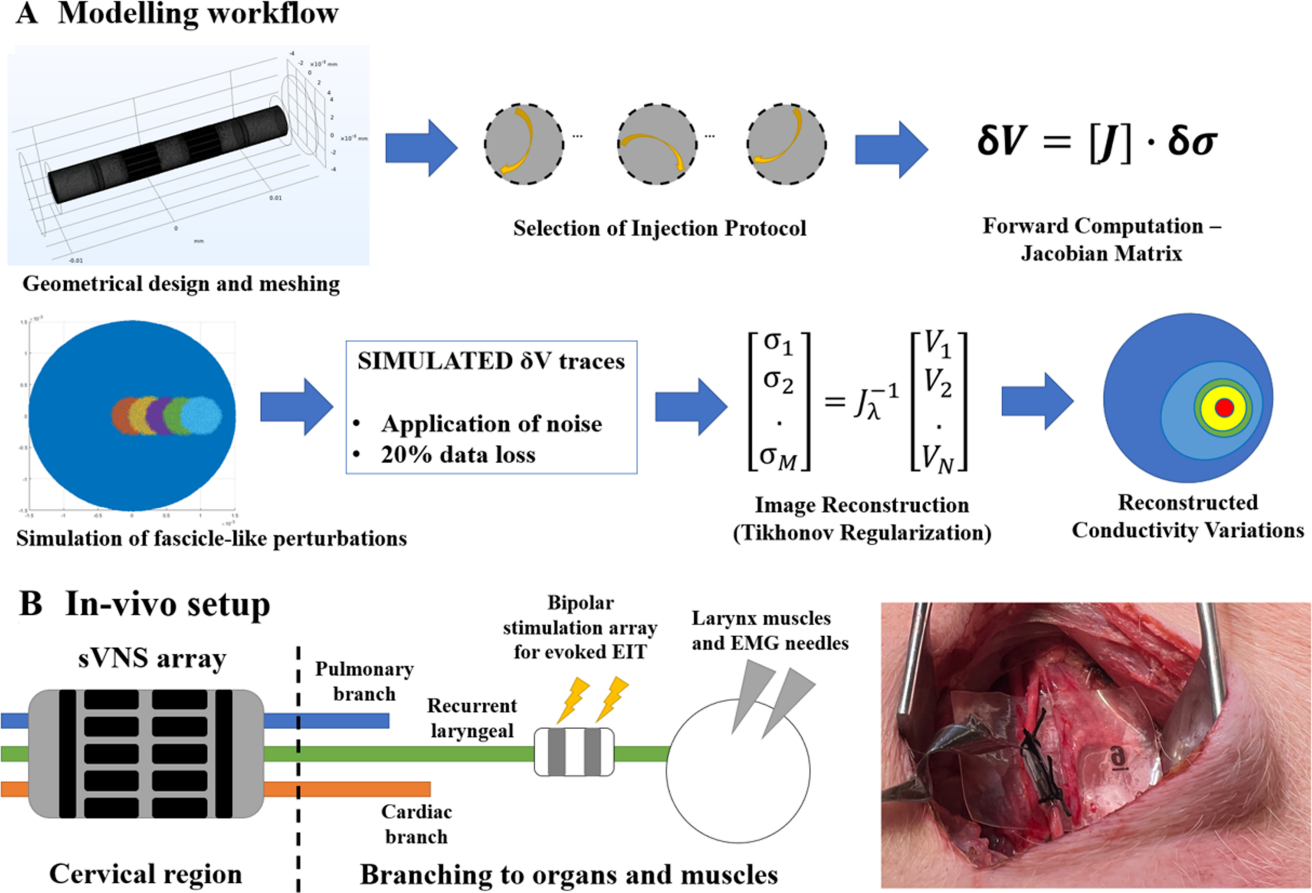
(**A**) Modelling workflow. A Jacobian matrix was computed from a given geometry, mesh and set of injection pairs. The Jacobian was used to generate simulated conductivity perturbations, retrieve corresponding voltages and perform image reconstruction. (**B**) In-vivo experimental setup. An sVNS nerve cuff was wrapped around the vagus nerve of a pig at the cervical level. A bipolar stimulation cuff was placed around the recurrent laryngeal branch for performing evoked-activity EIT, while EMG needles were placed at the level of the larynx. Image on the right shows application of the device on the vagus nerve in a pig.

The overall process used for modelling nerve EIT imaging (Figure 2, top) is similar to the one used in our previous work (7); models for the EIT, sVNS and Intercalated cuff designs were generated by creating a cylindrical representation of the nerve with a diameter of 3 mm, with electrodes wrapped around its circumference. Models were converted for forward computation into tetrahedral meshes with maximum element size of 40 µm and 100 µm for electrode surface and nerve volume, respectively. Meshing was performed in COMSOL Multiphysics (COMSOL Inc., USA). Forward problem solution was performed using the PEITS solver (15) which is based on the Complete Electrode Model (CEM) (16). Resulting tetrahedral meshes had ≈2.44 M, 3.3 M and 1.4 M elements, respectively. For image reconstruction, tetrahedral meshes were converted into simplified hexahedral (i.e., voxel-based) meshes with a voxel size of 150 µm and with 157 K, 143 K and 125 K elements, respectively.

Computation of forward problem solution was performed using the UCL PEITS fast parallel forward solver which implements a Complete Electrode Model (CEM) (15). The contact impedance for the CEM was set to 600 Ω for the EIT cuff geometry, a value compatible with our previous experimental work using this geometry (5). For the sVNS and Intercalated cuff, contact impedance was scaled to 300 Ω and 1040 Ω, respectively, based on the surface area of electrodes. The amplitude of injected current was set to 150 µA for all models. All simulations were performed using a skip-5 injection pattern as in our experimental pig vagus nerve work (5).

The sVNS array design was tested in a single-reference configuration, in which voltages were measured with respect to a reference electrode placed on a single side of the main circular array, e.g., right side. The EIT array design and the Intercalated design were tested in a double-reference configuration, with two reference electrodes electrically connected and placed symmetrically on both sides of the main array. For the Intercalated design, in order to minimise surface occupation over the nerve, individual dedicated reference rings as present in the sVNS design were replaced by using the sVNS electrodes in the shunted configuration as a reference. Electrodes used for measurements are shown in grey red pads in Figure 1, while reference pad/pads are shown in grey.

The background conductivity of the nerve model was 0.3 S/m. For each geometry under test, five perturbations of constant 20% nerve diameter were applied at 0, 250, 500, 750, and 1000 µm from the centre to simulate the activation of nerve fibres in a middle-sized fascicle. Activation of a fascicle at the given location was simulated by a 0.125% increase in background conductivity at the selected coordinates. The amplitude of the conductivity perturbation was chosen to reproduce the signal amplitude levels measured experimentally from the recurrent laryngeal branch of the pig vagus nerve (5). In this previous study, the signal-to-noise ratio (SNR) value of 4.1 ± 1.5 for laryngeal EIT impedance changes was obtained from peak signal amplitudes of ≈2 µV over a noise level of ≈0.5 µV (dataset available at https://discover.pennsieve.io/).

The resulting sets of simulated voltage variations (“δV”) collected for each model under test were subject to two steps aimed at making our assessment more realistic:

1. Random Gaussian noise was applied to each δV with an amplitude of 1 µV, compatible with realistic EIT noise in pig vagus nerve studies in the fast neural EIT bandwidth (5).
2. A random subset of 20% of δV traces was removed before image reconstruction to simulate data loss due to faulty electrodes, corrupted data, etc. The list was pre-generated *in-silico* with a pseudo-random process and was the same for each geometry.

Image reconstruction was performed using 0th-order Tikhonov regularization and noise-based voxel correction (7, 10, 17). The following markers were chosen as figures of merit to assess each design:

• **δσ_AVG_ [-]**—Amplitude of reconstructed conductivity change computed from top 50% voxels for each slice.
• **ECoM [µm]—**Centre-of-mass (CoM) error; localization error over the cross-section between the centre-of-mass of the reconstructed perturbation, computed from the intensity of the top 50% voxels, and the true location of the simulated perturbation.
• **CI [-]**—Circularity Index; a value in the range [0–1], with 1 being a perfect circle. This metric has been previously used to assess image deformation in EIT image reconstruction (12) when perturbation are perfectly circular in nature.

Metrics have been assessed individually for each design and perturbation, over the longitudinal direction of the nerve, for each cross-sectional slice contained below the nerve cuff. Afterwards, they have been averaged over perturbations for each design.

### 2.3 . *In vivo* recordings

EIT data was collected for experimental confirmation using the simplest electrode configuration identified during the *in-silico* stage, which was the sVNS geometry. The sVNS geometry cuff was manufactured using a process previously reported by our group (12) and used in the original sVNS study in sheep (4). Within this process, cuff electrode arrays are manufactured by encasing laser-patterned stainless steel electrodes in silicone; electrodes are coated with conductive polymer PEDOT to reduce contact impedance.

Data was collected with the paradigm previously developed for performing evoked EIT recordings at the level of the cervical vagus nerve from repeated stimulation of the recurrent laryngeal fascicle in pigs (5) (Figure 2, bottom). Briefly, in evoked-activity EIT, an additional bipolar stimulation cuff is placed downstream from the full vagus nerve around a specific fascicle, post-branching, to repeatedly evoke action potentials in fascicles with no spontaneous cyclical activity. Imaging is performed with the main EIT cuff at the level of the full vagus nerve.

Data was collected in three anaesthetised domestic pigs of ≈50 kg weight, during an on-going neuromodulation study. Surgery was performed to expose a segment of the left and/or right vagus nerves and allow placement of the electrode array. An electrical ground and earth electrodes were inserted into the surgical field. The recurrent laryngeal nerve from the same side of the exposed vagus nerve, i.e., left or right, was also exposed and a bipolar stimulating electrode (CorTec GmbH, I.D. 1.2–2 mm) was placed around it. EMG needles were implanted into the laryngeal muscle to record the laryngeal effects of selective VNS. All experimental procedures were ethically reviewed and carried out in accordance with the Animals (Scientific Procedures) Act 1986.

EIT was performed at a frequency of 6 kHZ, and a current amplitude of 200 µA applied sequentially to a pair of electrodes in skip-5 configuration for 30 s; as there were 14 electrodes, this yielded a total measurement time of 7 min. During EIT, the recurrent laryngeal nerve was stimulated with biphasic current pulses at 2 mA amplitude, 50 µs pulse width, and 20 Hz repetition frequency. The ScouseTom EIT system was used to perform stimulation, EIT, and record EMG data. A custom battery-powered current source (18) was employed for EIT recordings to ensure lowest possible noise levels. Recorded EIT traces were bandpass-filtered at ±1 kHz around the 6 kHz carrier frequency and demodulated by computing the absolute value of the Hilbert transform. Traces then underwent baseline subtraction and coherent averaging to increase SNR. Traces were then highpass-filtered at 100 Hz to remove possible contamination by movement artefacts.

EIT traces from all recordings at peak impedance change were tested for statistical significance (*T*-test against zero mean) and SNR was computed and compared with that of our previous study with separate sVNS/EIT cuffs. For nerves for which sVNS-based EMG recordings were available, image reconstruction was performed from EIT traces using the same noise-corrected 0th-order Tikhonov process as the simulations; the CoMs from sVNS and EIT were then compared as additional mean of validation.

For sVNS, CoM was computed by identifying the electrode pairs with EMG response to sVNS stimulation, which was considered the closest pairs to the location of the recurrent laryngeal fascicle. Based on the previous sVNS study (4), it was assumed that at the optimal selective current amplitude activated fibres in the outer 2/3 of the nerve radius and did not activate deeper regions. Thus, CoM was computed from the average angular position of the responsive pairs and assumed a maximum density of sVNS current at ≈2/3 of the nerve's radius.

For EIT, CoM was computed over the location of the 16 voxels (i.e., 4 × 4 grid) showing the highest conductivity variation over the cross-section of the nerve at the time of peak EIT signal.

## 3. Results

### 3.1. Modelling results

**Figure 3:**
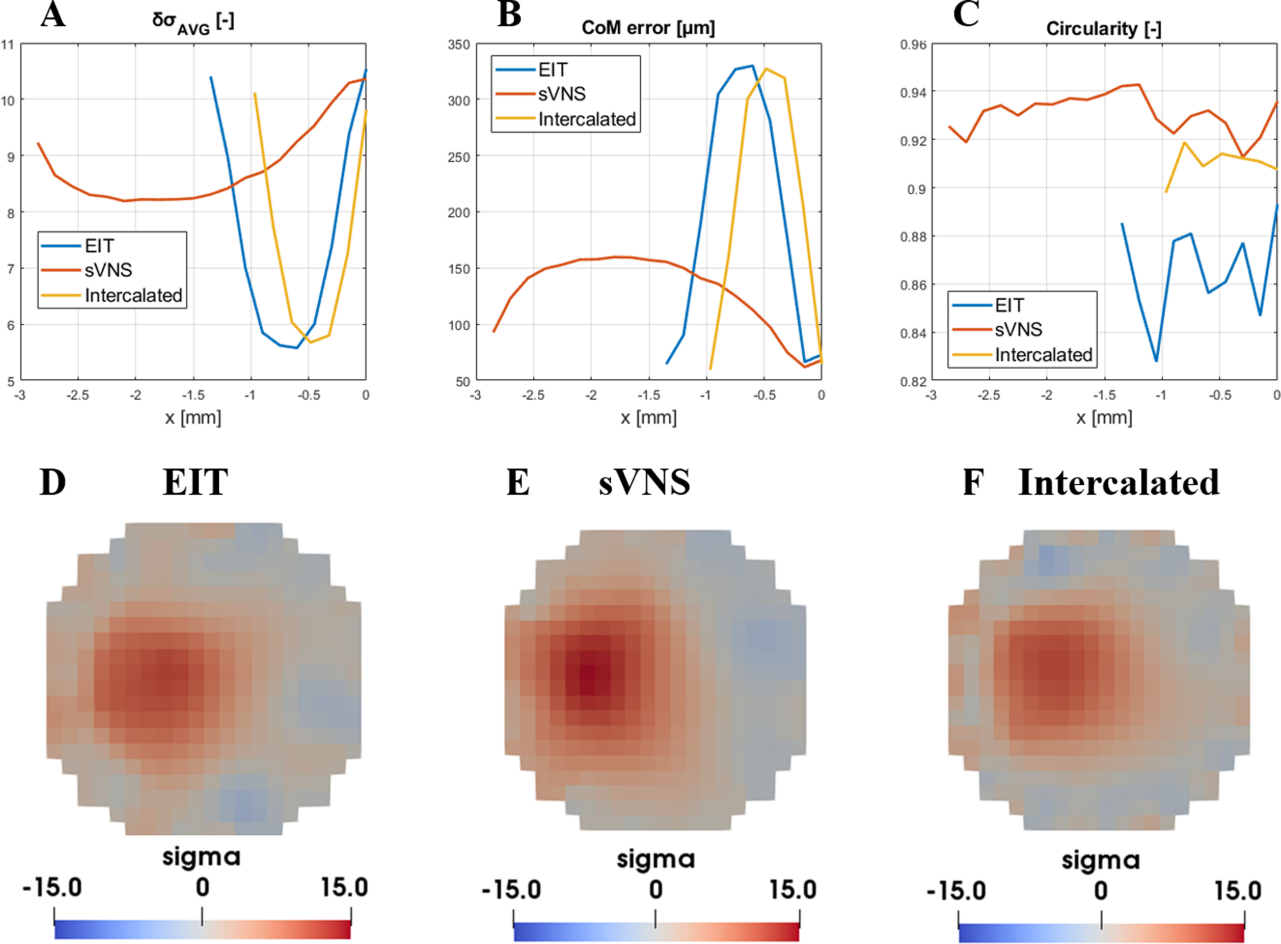
Evaluation of figures of merit ((**A**) δσ_AVG_, (**B**) CoM and (**C**) circularity) for the EIT, sVNS and intercalated array geometries. For each geometry, circumferential nerve area below the electrodes is divided into multiple cross-sections with single-voxel resolution and figures of merit are evaluate over each one along the longitudinal direction. For this reason, geometries with longer electrodes such as the sVNS design have longer profiles. (**D–F**) Image reconstruction results for each geometry at the location of maximum signal intensity and lowest CoM localisation error, identified by data in top row. Images are shown for the perturbation centred at 500 µm from the centre and with a scale representative of the maximum value reached across all images.

*δσ_AVG_*; the EIT, sVNS and Intercalated geometries (Figure 3) showed similar top performance for all the array designs in terms of δσ_AVG_, which can be considered a surrogate of SNR at the image level. δσ_AVG_ was in the range of 9.8–10.5, corresponding to a ≈7% difference between the lowest performing design (Intercalated) and the best performer (EIT design).

*CoM error*; in terms of CoM localisation error, all geometries reached the lowest CoM error in the range of 64.4–72.9 µm, corresponding to a 13% maximum difference between designs.

*Circularity*; the sVNS design was the best performer with the highest circularity value of ≈0.94, compared to ≈0.91 of the Intercalated design and ≈0.87 of the original EIT design, corresponding to ≈8% difference between designs.

Our objective criteria for evaluating whether one approach was superior to the other or not were a difference >10% in δσ_AVG_, CoM error or circularity. For CoM error, any difference >10% but inferior to the voxel size of 150 µm routinely used for vagus nerve EIT would be considered negligible.

Based on these criteria, the results of this *in-silico* analysis showed that all design achieved equal imaging performance. However, in these conditions of equal performance, the sVNS design has the added value of allowing surgical implantation of only one cuff with the lowest number of electrodes. Thus, the sVNS design was designated as the ideal geometry for EIT imaging on the vagus nerve, with image analysis to be performed on the cross-section closest to the reference ring. This design was used for *in vivo* testing to confirm modelling results.

### 3.2. Results from *in vivo* recordings

EIT recordings were performed on a total of *N* = 4 nerves from three pigs, from two left and two right vagi. Laryngeal EMG recordings performed during sVNS for validation were available from a subset of *N* = 2 nerves, one left and one right vagus.

Over the entire dataset (*N* = 4), recorded *δ*V traces had an average value of 1.35 ± 0.52 µV and were statistically significant (*p* = 0.0142). Average SNR was 3.9 ± 2.4, a value compatible with our previous finding of 4.1 ± 1.5 from the previous study with separate cuffs (5).

**Figure 4:**
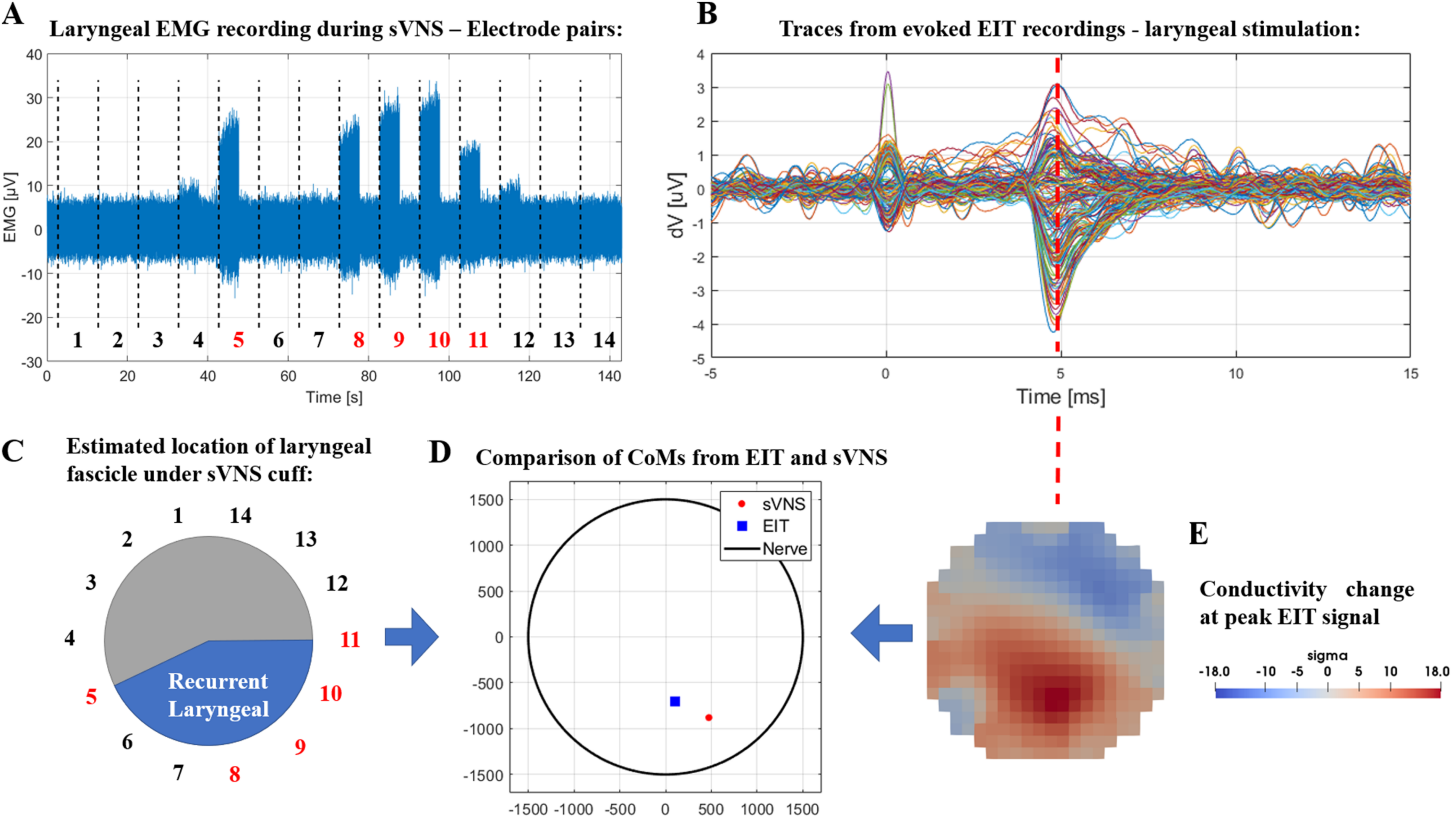
(**A**) Laryngeal EMG traces from sVNS of the cervical vagus nerve. (**B**) Vagus nerve EIT traces from evoked stimulation of the recurrent laryngeal branch. (**C**) Estimated location of the recurrent laryngeal fascicle inside the cervical vagus nerve as identified by sVNS/EMG (**C**) or EIT (**E**). (**D**) Centers-of-Mass identified from both techniques are overlayed in the lower central panel.

In the two nerves with EMG recordings available, data from EMG needles placed in the larynx during sVNS stimulation of the cervical vagus showed activation in response to stimulation of localized groups of 5–6 electrode pairs. CoMs computed from sVNS-EMG data and EIT data were localised in the same region of the cross-section of the nerve. Figure 4 shows a complete example of this process for one nerve. The co-localisation error with the CoM was 433 ± 31 µm in terms of cartesian distance (≈14% of nerve diameter for an average 3 mm vagus nerve), separable into a radial component of 352 ± 88 µm (≈12% of nerve diameter) and an angular component of −24 ± 6° (≈13% of max error of 180°). This accuracy value is also compatible with our previous findings.

## 4. Discussion

### 4.1. Modelling results

In this study, different geometrical designs for electrode arrays were assessed to perform EIT imaging of neural activity when embedded in a nerve cuff. The state-of-the-art solution for this problem was to implant separate and adjacent arrays for sVNS and EIT imaging. The EIT array geometry from this setup with separate EIT/sVNS cuffs was compared with the direct use of the existing electrodes on the sVNS cuff for EIT and with the design of a new array combining intercalated electrode rings for sVNS and EIT.

While it is theoretically possible to come up with many more designs, the optimal geometry for sVNS in the vagus has already been found (4) and the problem scope was limited specifically to integrating EIT on the same cuff.

Results of the *in-silico* analysis of geometries returned comparable performance; however, the existing sVNS design has the added values of not requiring the implantation of two different cuffs, reducing the surgical burden, and does not have an additional set of electrodes as does the Intercalated design, resulting in simpler manufacturing. Thus, our analysis determined that the existing sVNS design could be used for EIT recordings.

The markers chosen to evaluate imaging performance represent the most important features for nerve EIT imaging: high signal SNR in the reconstructed image, accurate localization of the centre of a fascicle, and no shape deformation. Arguably, low CoM error is the most important feature as nerve EIT imaging is primarily targeted at informing sVNS without a trial-and-error procedure.

All three designs showed a CoM localisation error inferior to the size of a single voxel in our reconstruction mesh. While decreasing voxel size is theoretically possible and could allow for a more accurate comparison, there is a trade-off involved as the inverse problem solved during image reconstruction would have more elements to solve for, while the amount of independent information is fixed by the number of electrodes and drive pattern. Thus, we consider the current voxel size of 150 µm over a 3 mm diameter as a reasonable choice.

Circularity, while conceptually only a meaningful marker for circular perturbations, was chosen as a surrogate to assess the capacity of different geometries to maintain the original shape of the perturbation during image reconstruction; we expect the results from the CI index to hold reasonably true for more complex shapes.

### 4.2. Experimental validation

Our *in-silico* results were validated experimentally by performing EIT imaging of evoked activity on the left and right vagi of pigs. In previous studies (5, 8), segmented MicroCT scans of the nerve fascicles were used as the gold standard to validate EIT results; in this study, only sVNS results are employed, and only for a single animal. While this is technically a limitation, our previous study already established a correlation between laryngeal sVNS and the recurrent laryngeal fascicle. More so, from a physio-anatomical perspective, larynx EMG activation can only be a consequence of laryngeal sVNS and evoked FN-EIT is performed through a dedicated bipolar cuff that only stimulates the recurrent laryngeal fascicle.

To perform evoked-activity EIT, the recurrent laryngeal branch of the vagus nerve was stimulated continuously for 7 min with pulse amplitude of 2 mA, pulse width of 50 µs and repetition rate of 20 Hz. These parameters were chosen to ensure supramaximal stimulation leading to complete fiber recruitment and maximal impedance changes detected by EIT. While similar parameters were chosen in previous studies (5, 8) which led to successful correlation of EIT images with reference techniques, further investigation is needed to confirm that nerve response does not decrease over this time.

In terms of experimental results, the recording performed in this study achieved an SNR of 3.9 ± 2.4; this is comparable to our previous study in which evoked laryngeal FN-EIT had an SNR of 4.1 ± 1.5, suggesting that alteration to cuff geometry did not reduce signal quality.

In contrast, EIT imaging achieved a CoM co-localisation error of ≈14% of nerve diameter in this study compared to sVNS data, which is lower than the 25% achieved vs. MicroCT data in our previous study. While this could be an early indicator of the co-localisation advantage granted by the use of a single cuff, the reference techniques are different and the new geometry has been tested on only two nerves; further experimental EIT recordings with the sVNS geometry are needed to confirm the co-localisation improvement. In summary, our experimental results validated the *in-silico* prediction that the sVNS array can be also used for EIT recordings.

The use of sVNS array geometry for EIT imaging was investigated for evoked activity only; however, there is no reason to assume imaging of spontaneous activity cannot be performed in this configuration, as the mechanism driving evoked and spontaneous FN-EIT is conceptually the same. As a consequence, targeted neuromodulation of the vagus nerve becomes a possibility: a future sVNS/FN-EIT implanted device will be able to identify the electrodes closest to a specific fascicle, e.g., the cardiac fascicle, using spontaneous FN-EIT and then perform targeted sVNS on the same electrodes.

### 4.3. Answer to specific questions and future work

It is now possible to answer the questions we set up at the beginning of the study:

• Is it possible to develop a combined sVNS/EIT nerve cuff without compromising on stimulation or imaging features?
  **YES**—Imaging and stimulation features can co-exist without any loss of performance.
• What is the simplest geometry for a combined sVNS/EIT cuff?
  The simplest geometry is the original **sVNS cuff** design. It has similar imaging performance as the other tested designs but requires lower effort for manufacturing and surgical implantation.

Future work in vagus nerve EIT will focus on different areas such as: expanding to subdiaphragmatic branches of the vagus, moving to human trials, and developing an implantable version of the hardware.

## Data Availability

The raw data supporting the conclusions of this article will be made available by the authors, without undue reservation.
